# Evaluation of postoperative results after a presurgical optimisation programme

**DOI:** 10.1186/s13741-024-00430-7

**Published:** 2024-07-15

**Authors:** Francisco García Sánchez, Natalia Mudarra García

**Affiliations:** 1 Surgical Prehabilitation Unit, Infanta Cristina University Hospital., Avenida 9 de Junio 2. Parla., Madrid, 28981 Spain; 2 IDIPHISA. Medical Department. Faculty of Medicine, University Complutense of Madrid, Madrid, Spain; 3 IDIPHISA. Nurse Department. Faculty of Nurse. University Complutense of Madrid, Madrid, Spain

**Keywords:** Presurgical optimisation programme, Preoperative optimisation, Major surgery, Postoperative complications

## Abstract

**Background:**

Presurgical optimisation programmes decrease the risk of postoperative complications, reduce hospital stays and speed up patient recovery. They usually involve a multidisciplinary team addressing physical, nutritional and psychosocial issues. The objective of this study was to assess the results of implementing a presurgical optimisation programme led by a liaison nurse in patients undergoing major surgery in a primary general hospital.

**Methods:**

An observational, retrospective, descriptive, cross-sectional, comparative study based on the revision of patients’ health records undergoing major surgery between January 2019 and December 2022. Patients entering the presurgical optimisation programme (intervention group) were compared with patients receiving usual medical care (control group). The presurgical optimisation programme consisted of oral nutritional supplementation, physical exercise, strengthening of lung capacity and psychological and emotional support. Frequency (%) of surgery complications and use of healthcare resources (duration of hospitalisation, time spent in the intensive care unit (ICU), and readmission) at day 30 were recorded. Descriptive statistics were applied.

**Results:**

Two hundred eleven patients (58.5% men, mean age: 65.76 years (SD 11.5), 75.2%. non-smokers; mean body mass index (BMI): 28.32 (SD 5.38); mean Nutritional Risk Score (NRS) 3.71 (SD 1.35; oncology diagnosis: 88.6%) were included: 135 in the intervention group, and 76 in the control group. The average duration of the presurgical optimisation programme was 20 days (SD 5). Frequency of postoperative complications was 25% (*n* = 33) in the intervention group and 52.6% (*n* = 40) in the control group (*p* < 0.001) [odds ratio (OR) = 3.4; 95% confidence interval (CI) (1.8; 6.2)]. 14.5% (*n* = 19) of patients in the intervention group and 34.2% (*n* = 26) in the control group had remote postoperative complications [OR = 3.1; 95% CI (1.6; 6.2)]. Patients in the intervention group spent fewer days in the hospital [mean 8.34 (SD 6.70) vs 11.63 (SD 10.63)], and there were fewer readmissions at 30 days (7.6% vs 19.7%) compared with the control group.

**Conclusions:**

A presurgical optimisation programme led by a liaison nurse decreases the rate of immediate and late surgical complications and reduces hospital stays and readmissions in patients undergoing major surgery.

**Supplementary Information:**

The online version contains supplementary material available at 10.1186/s13741-024-00430-7.

## Introduction

Presurgical optimisation programmes are used before surgery to decrease the risk of postoperative complications, reduce hospital stays and speed up patient recovery (Vía 2021; Lawrence et al. 2004). Although not standardised, these multidisciplinary interventions usually include physical, nutritional, and psychosocial conditioning (Baimas-George et al. 2020) to improve the functional capacity of patients, control comorbidities, and adjust nutrition. The haemoglobin levels are also checked and optimised (in cases of anaemia), and appropriate steps are taken to reduce the anxiety and stress levels of the patient (Calleja et al. 2016; Peters et al. 2018). Such programmes are cost-effective because they can reduce postoperative complications and shorten hospitalisation, thus decreasing the use of medical care resources while improving the patient’s health (Leeds et al. 2021).

Presurgical optimisation programmes differ in length, indicated nutritional supplementation, number of visits before surgery and the participating professionals (Hijazi et al. 2017). Programmes in the United Kingdom and the Netherlands involve up to ten specialists (Davis et al. 2022). Each specialist assesses the patient and proposes the procedures to be followed before the surgery (Davis et al. 2022). Such programmes are complex and should be adapted to the needs of each individual; this implies logistical challenges. Nursing professionals usually coordinate the process and accompany the patient during the programme and the postoperative period. They do not intervene in the clinical or care-needs assessment of the patients (Dana et al. 2022).

Several studies have shown improvements in clinical outcomes and the overall health status of patients participating in presurgical optimisation programmes (Khadem et al. 2023). The reported examples include patients undergoing abdominal surgery for gastrointestinal, urological, gynaecological, hepatobiliary, or pancreatic malignancies. It has been shown that participation in these programmes can correct myopia or sarcopenia associated with poor prognosis. Moreover, surgical prehabilitation improves the tolerability of oncospecific treatments and the health-related quality of life (Martínez-Ortega et al. 2022).

Achieving good nutritional status is a fundamental component of presurgical optimisation programmes (Matthews et al. 2021) as it is one of the most important factors determining the results of surgical intervention (Ho et al. 2015). Malnutrition in surgical patients prolongs hospital stays and increases the use of resources necessary for the care and treatment of complications. It is also associated with delayed wound healing and a rise in hospital readmissions (Thomas et al. 2016; Mosquera et al. 2016). Moreover, malnutrition is a predictive factor for mortality, impaired mobility and increased long-term dependency (Helminen et al. 2017; Koren-Hakim et al. 2016). The European Society for Clinical Nutrition and Metabolism recommends that all malnourished patients or those at risk of malnutrition receive nutritional support for 7 to 14 days before the intervention and during the postoperative period (Weimann et al. 2021). Sometimes, nutritional improvement is needed within a short time, such as in cancer patients undergoing surgical resection of a solid tumour. In such cases, the diet must incorporate proteins of high biological value and certain amino acids (e.g., leucine). When combined with resistance exercise, this treatment can synergistically affect muscle tissue, enhancing protein synthesis and preventing sarcopenia (Rubio del Peral and Gracia Josa 2019).

Around 10% of patients undergoing abdominal surgery in Spain suffer from postoperative complications (Alastrué 2023) associated with increased mortality, prolonged hospital stays, and rising healthcare costs (Canet et al. 2010; Gili-Ortiz et al. 2015). The objective of the study was to assess if implementing a presurgical optimisation programme led by a liaison nurse reduces post-surgery complications and length of hospital stay in the intervention group in usual clinical practice. The evolution of anaemia and nutritional status of the participating patients were monitored at the start of the programme and 30 days after the surgery.

## Materials and methods

### Study design

**Fig. 1:**
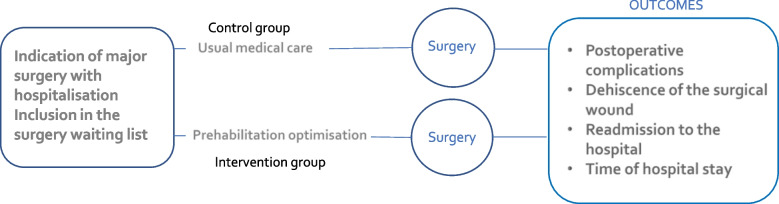
Study diagram

This observational, descriptive, comparative, cross-sectional study was carried out at the Infanta Cristina University Hospital in Parla (Madrid). The presurgical optimisation programme, led by a liaison nurse, was evaluated by comparing the intervention group (patients undergoing the clinical, functional, and psychological optimisation) with those who received the usual preoperative care (without clinical optimisation; control group). All patients in the study underwent major elective surgeries (Fig. 1).

Patients over 18 years old, awaiting major elective surgery and admitted to the oncology or traumatology hospital departments, were included. Medical records of eligible patients undergoing major elective surgery between January 1, 2019, and December 30, 2022, were retrospectively reviewed. Exclusions comprised patients referred elsewhere, requiring acute care surgery, or experiencing cancelled surgeries.

Recruitment was consecutive in both the intervention and control groups. However, due to the interruption in the presurgical programme caused by the COVID-19 pandemic, the intervention group included the patients who participated in the programme and had surgery during the years 2020, 2021 and 2022. The control group consisted of patients who underwent surgery in 2019 (Fig. 1).

The presurgical programme was introduced at the hospital in 2020. All patients on the surgery waiting list went through the surgical optimisation programme except those who did not meet the inclusion criteria, underwent emergency surgery, or were hospitalised in the otorhinolaryngology department.

### Study variables

The following sociodemographic and clinical variables were collected: sex, age, smoking habits, body mass index (BMI), diagnosis of oncological disease (yes or no), type of oncological or non-oncological diagnosis, comorbidities, diabetes mellitus status (adequately controlled or not), immunocompromised condition (yes or no), the recommendation for neoadjuvant therapy (yes or no), and ascites (present or not). The type of surgical procedure was also recorded.

The description of clinical evolution after surgery included the need for conversion. This was defined as the change of the surgical procedure from laparoscopy to laparotomy. Other factors or events recorded (details shown in Table 3) were complications, type of postoperative infectious complications stemming from the surgery, pulmonary complications, intra-abdominal complications, paralytic ileus, suture dehiscence, wound complications, post-stress haemorrhage, whole blood transfusion requirement, number of units of red blood cells transfused, multiple organ failure, remote complications defined as complications 30 days after surgery and death.

Additionally, in the intervention group, the values for haemoglobin, ferritin, albumin, prealbumin, and C-reactive protein (CRP) levels were taken, as well as total lymphocyte count and total cholesterol. This was done to determine the nutritional and inflammatory status of the patient at the start of the presurgical optimisation programme, the day before surgery (i.e., 21 to 30 days from the admission to the programme) and 30 days after surgery.

To assess the use of resources after surgery, the following data were recorded: the duration of hospitalisation, time spent in the intensive care unit (ICU) due to emergency admission due to complications, and readmission (yes or no; 30 days after the surgery). Any reinterventions were also registered (yes or no), defined as new surgeries related to the initial intervention due to poor evolution or complications.

### Intervention

**Fig. 2:**
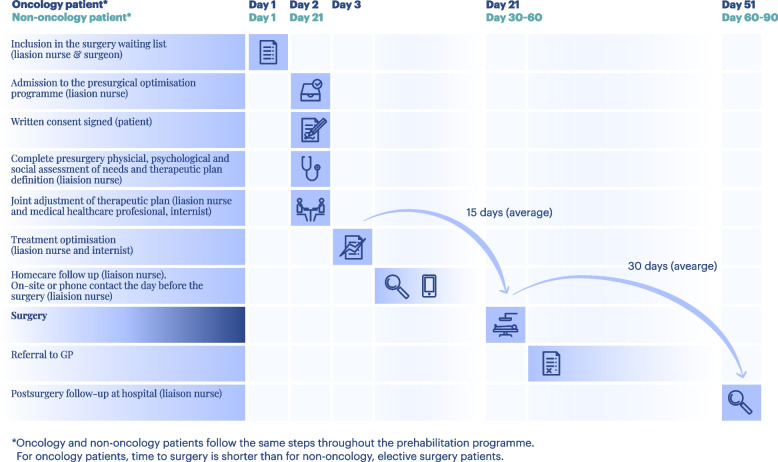
Presurgical optimisation programme flow: oncology vs non-oncology patients

The intervention group followed the protocol for the presurgical optimisation programme for 15–30 days (Fig. 2). The presurgical optimisation program initiates with the patient’s inclusion on the surgery waiting list by the attending surgeon. Cancer patients undergo evaluation by the liaison nurse within 72 h of their surgery request, with surgery scheduled within 30 days (Fig. 2). Non-cancer patients, whose surgery waiting period extends to 2 to 3 months, are assessed 21 to 30 days after listing.

Once the patient enters a presurgical optimisation programme, a comprehensive assessment is performed. This includes screening using the Nutritional Risk Score (NRS) 2002 (Kondrup et al. 2003) and the Global Leadership Initiative on Malnutrition criteria (GLIM) (Kondrup et al. 2003) to establish the nutritional status and choose the most appropriate supplement regimen for each patient. Laboratory tests are conducted to detect anaemia (haemoglobin level < 13 g/dL in a peripheral blood test). Anthropometric (weight, height, BMI, and the diameter of the rectus femoris muscle) and other (fat, muscle, and water mass) parameters are also obtained. Nutritional ultrasound analysis is performed to assess body composition. The functional status is examined using a gait test for aerobic resistance (Crapo et al. 2012) and dynamometry to determine muscle strength (Stark et al. 2011). Barthel scale is employed to define the degree of patient dependency (Mahoney and Barthel 1965). Lung capacity is estimated using incentive spirometry (Carey et al. 2014).

Moreover, a psychological evaluation is carried out to measure the self-esteem of the patient (Rosenberg scale) ( American Psychological Association^ 1965^). The body image questionnaire (Hopwood et al. 2001) is employed to determine the perception of body image. The EQ-5D-5L survey is used to obtain health-related quality of life scores (Herdman et al. 2001). The usual pharmacological treatment and the degree of adherence are reviewed for each patient. Finally, the ability of the patient to understand the information pertaining to the objectives, implications, and the need for inclusion in the programme is assessed. Based on the obtained results, the procedures are adapted to the individual needs of each patient (Table 1).

**Table 1:** Summary of the presurgical optimisation programme characteristics (intervention group)

Assessment	Tool	Cut-off points that define the patient’s needs	Optimisation considered
Nutritional assessment and inflammation	Laboratory variables (blood test) NRS 2002	NRS 2002 > 3 (Thoresen et al. 2013)^a^	Supplementation with deficient vitamins and minerals
	GLIM criteria	> 1 phenotypical point + > 1 etiological point (Cederholm et al. 2019)^b^	NOS supplementation
Detection of anaemia	Laboratory variables	Haemoglobin < 13 g/dL (men) and < 12 g/dL (women) (Cappellini and Motta 2015 Oct)	Ferric carboxymaltose supplementation
Nutritional assessment	Anthropometric variables: • Weight • Size • BMI • Nutritional ultrasound (preperitoneal fat)	BMI > 25 (World Health Organization 2024)^c^ Preperitoneal fat (> 0.8 cm) (García-Almeida et al. 2023)^d^	Hyperproteic and hypercaloric supplementation
Diet history for the 3 preceding days	Structured form	-	Diet adjustment
Assessment of fat, muscle and water mass	Bioimpedance	Visceral adipose tissue measured by impedancemetry > 12% (73]^e^	Diet adjustment
Functional state assessment	Dynamometry test		Physical activity, a minimum of 150 min of aerobic exercise weekly
	Gait speed test of 4 m and physical activity Measurement of the area of the rectus femoris muscle Barthel scale	Pathological: Gait test ≤ 0.8 m/s (Studenski et al. 2014)^f^ Area of rectus femoris muscle < 1 cm^2 (^García-Almeida et al. 2023^)g^ Barthel score < 100 (Mahoney and Barthel 1965)^h^	
Lung capacity assessment	Incentive spirometry	There are no cut-off points; the aim is to improve the initial value	Strengthening through incentive spirometry, raising the lung capacity to 500 ml above their baseline level before surgery
Psychological assessment	Questionnaire on the perception of body image and physical activity		Referral to psychologist
	Psycho-oncological scale (Rosenberg)	< 15: low self-esteem (García et al. 2019)^i^	
Quality of life	Euro-QoL5D	> 5 points (Herdman et al. 2001)	
Medication habitually taken by the patient and degree of adherence	Structured form	Assessment of the form completed by the nurse; no defined cut-off points	Medication adjustment to achieve high adherence to the pharmacological treatment to improve comorbidity control
Assessment of the understanding of the medical information received	Structured form	Assessment of the form completed by the nurse	Explanation of the expected benefits of the programme

*GLIM* Global Leadership Initiative on Malnutrition, *BMI* Body mass index, *NRS* Nutritional risk score, *NOS* Nutritional oral supplement

^a^NRS-2002 score: total number of points ranges from 0 to 7. Patients with a score ≥ 3 indicates that a patient is at risk of malnutrition

^b^The GLIM includes three phenotypical criteria (weight loss, low BMI, and reduced muscle mass) and two etiological criteria (reduced food intake or absorption, and increased disease burden or inflammation). Malnutrition diagnosis requires at least 1 phenotypic criteria and 1 etiologic criteria. The severity of malnutrition is determined based on phenotypic criteria following thresholds published by Cederholm et al. (2019)

^c^BMI > 25 indicates overweight

^d^Preperitoneal fat > 0.8 cm measured by ultrasound indicates cardiovascular risk

^e^Visceral adipose tissue measured by bioimpedance: < 12% healthy proportion of visceral fat > 12% excessive proportion visceral fat

^f^Gait test ≤ 0.8 m/s is an indicator of sarcopenia

^g^Area of rectus femoris muscle < 1 cm^2^ is an indicator of sarcopenia

^h^Barthel score: 100 points indicates complete independence to perform basic activities of daily living, < 100 points indicates dependency (91–99: mild, 61–90: moderate, 21–60: severe, ≤ 20: total dependence)

^i^Rosenberg scale (scale ranges from 0 to 30): 15–25 indicates normal range and < 15 suggest low-self-esteem

The nutritional status was optimised if the Nutritional Risk Score (NRS) 2002 of the patient was greater than 3 points (considering the anthropometric parameters). To achieve this, two hyperproteic and hypercaloric oral supplementation, containing 20.8 g of protein (100% lactoprotein serum), leucine and vitamin D, were administered (Table S1 and Table S2). One to three bottles of 200 ml per day were recommended per patient. The diet was adjusted based on the amount of body fat, muscle and water mass of each patient and their diet during the preceding 3 days. Patients with anaemia were treated with ferric carboxymaltose.

The patients engaged in physical exercise comprising at least 150 min of weekly aerobic activity. Lung capacity was strengthened using the incentive spirometer to raise the patient’s lung capacity to 500 ml above their baseline level before surgery. Patients with low self-esteem (Rosenberg score < 25 points) and with a low score in their assessment of the quality of life (Euro-QoL5D > 5 points) were referred to a psychologist.

Control of comorbidities was improved by adjusting pharmacological treatments, complementing the usual treatment, and increasing adherence. Carbohydrate drinks were recommended up to 2 h before surgery to reduce the preoperative fasting time and to mitigate the transient increase in insulin resistance that elective surgeries often cause (Ho et al. 2015). Health education was undertaken to improve the understanding of the presurgery optimisation programme, objectives and expected outcomes; the process was adapted to the family, social and personal background of the patient.

#### Role of the liaison nurse in the presurgical optimisation programme

**Fig. 3:**
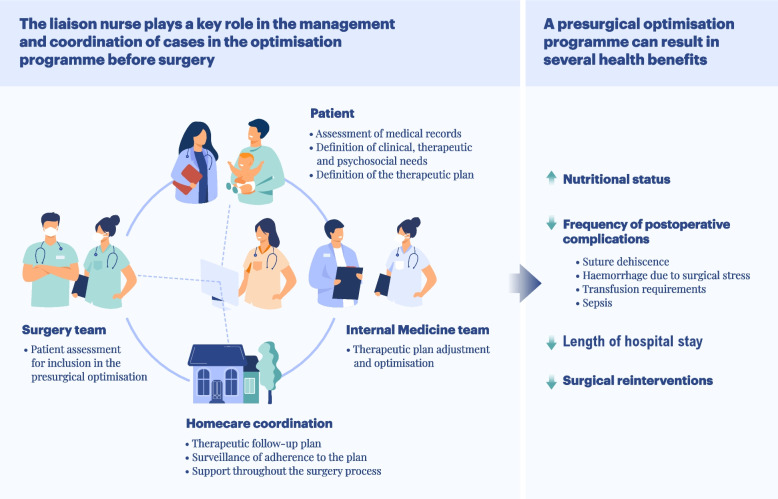
Presurgical optimisation programme: elements, structure, and results

The liaison nurse is responsible for coordinating and executing the proposed presurgical optimisation programme. The nurse conducts the assessments described in Table 2 and identifies patient needs. Based on the obtained results, the recommendations for individual optimisation are prepared, to be validated by the internist. In addition, the liaison nurse monitors the patients during the execution of the programme and performs a check-up on the day before the surgery and 30 days later (Fig. 3).

**Table 2:** Pre-surgery sociodemographic and clinical characteristics of the total population and the groups compared

		Total *N* = 211	Intervention group *N* = 135	Control group *N* = 76
Sociodemographic variables				
Mean age (SD)		65.67 (11.5)	64.43 (12.1)	67.88 (10.2)
Sex *N* (%)	Male *N* (%)	127 (58.6%)	73 (54.1%)	53 (69.7%)
	Female *N* (%)	84 (39.1%)	62 (45.9%)	23 (30.3%)
Smoker *N* (%)	< 10 cigarettes a day	12 (5.7%)	8 (5.9%)	4 (5.3%)
	> 10 cigarettes a day	20 (9.5%)	15 (11.2%)	5 (6.6%)
	> 20 cigarettes a day	21 (9.5%)	13 (9%)	8 (10.5%)
	Non-smoker	158 (75.2%)	99 (73.9%)	59 (77.6%)
Average BMI (SD)		28.11 (5.3)	27.58 (5.6)	28.64 (5.0)
Clinical variables				
Mean NRS (SD)		3.71 (1.4)	3.82 (1.5)	3.29 (0.5)
Cancer diganosis *N* (%)	Yes	186 (88.6%)	119 (88.8%)	67 (88.2%)
	No	25 (11.4%)	16 (11.2%)	9 (11.8%)
Type of cancer *N* (%)	Colorectal adenocarcinoma	118 (54.9%)	68 (50.4%)	50 (65.8%)
	Gastric adenocarcinoma	16 (7.4%)	10 (7.4%)	6 (7.9%)
	Ileocecal adenocarcinoma	5 (2.3%)	2 (1.5%)	3 (3.9%)
	Ampullary cancer	4 (1.9%)	1 (0.7%)	3 (3.9%)
	Prostate adenocarcinoma	3 (1.4%)	2 (1.5%)	1 (1.3%)
	Duodenal adenocarcinoma	3 (1.4%)	2 (1.5%)	1 (1.3%)
	Renal neoplasm	15 (7.0%)	15 (11.1%)	0
	Vesicular adenoma	1 (0.5%)	1 (0.7%)	0
	Gynaecological neoplasm	9 (4.2%)	9 (6.7%)	0
	Ureter	3 (1.4%)	3 (2.2%)	0
	Ovary	5 (2.3%)	5 (3.7%)	0
	Pancreatic carcinoma	5 (2.3%)	2 (1.5%)	3 (3.9%)
	Hepatocellular carcinoma	2 (0.9%)	1 (0.7%)	1 (1.3%)
	Neuroendocrine tumour	2 (0.9%)	0	2 (2.6%)
	Colon adenoma	9 (4.2%)	5 (3.7%)	4 (5.3%)
	Cholangiocarcinoma	4 (1.9%)	3 (2.2%)	1 (1.3%)
Non-cancer diagnosis *N* (%)	Ulcerative colitis	1 (0.5%)	1 (0.7%)	0
	Crohn's	2 (0.9%)	2 (1.5%)	0
	GIST	4 (1.9%)	3 (2.2%)	1 (1.3%)
Comorbidities *N* (%)	Heart disease	52 (24.8%)	44 (32.6%)	8 (10.5%)
	Diabetes	47 (21.2%)	26 (19.3%)	21 (27.6%)
	Lung disease	19 (9.0%)	8 (5.9%)	11 (14.5%)
	Renal disease	1 (0.5%)	0	1 (1.3%)
	Digestive system disease	4 (1.9%)	1 (0.7%)	3 (3.9%)
	Others	20 (9.5%)	19 (14.1%)	1 (1.3%)
	More than one	28 (13.3%)	21 (15.6%)	7 (9.2%)
	None	87 (41%)	42 (31.1%)	45 (59.2%)
Poorly controlled DM *N* (%)	Yes	19 (9.2%)	7 (5.3%)	12 (16%)
	No	192 (90.8%)	128 (94.7%)	64 (84%)
Immunocompromised *N* (%)	Yes	41 (19.5%)	32 (23.9%)	9 (11.8%)
	No	171 (80.5%)	103 (76.1%)	67 (88.2%)
Ascites *N* (%)	Yes	6 (2.9%)	5 (3.8%)	1 (1.3%)
	No	205 (97.1%)	130 (96.2%)	75 (98.7%)
Surgical procedure type *N* (%)	Laparotomy	92 (44.7%)	54 (41.2%)	38 (50.7%)
	Laparoscopy	119 (55.3%)	81 (58.8%)	38 (49.3%)
ICU admission *N* (%)	Yes	58 (27.9%)	28 (21.2%)	30 (39.5%)
	No	153 (72.1%)	107 (78.8%)	46 (60.5%)

*BMI* Body mass index, *DM* Diabetes mellitus, *GIST* Gastrointestinal stromal tumours, *ICU* Intensive care unit, *NRS* Nutritional risk score, *SD* Standard deviation

#### Data collection

All data were collected in a specially designed Excel sheet for this study by the researchers (N.M. and F.G.) through a review of patients’ medical records.

#### Ethical aspects

This study was approved by the Ethics and Research Committee of the Puerta de Hierro University Hospital (ACT 15.18).

The access to study files was password protected and restricted to the responsible researchers (N.M. and F.G.) according to current data protection regulations (BOE-A-2018, Regulation 2016).

### Statistical analysis

Assuming 15% losses, a 95% confidence level, 3% precision and 5% proportion, an appropriate sample size of 211 was estimated. Convenience sampling was performed.

All study variables were examined to assess their distribution. The categorical variables were described using the percentage associated with each possible response option, and the quantitative variables using the mean, standard deviation (SD), and range. For comparisons between variables and hypothesis testing, the chi-square test was employed for categorical variables, Student’s *t* test or ANOVA for quantitative variables that were normally distributed, and the Mann–Whitney *U* test or the Kruskal–Wallis test for quantitative variables without a normal distribution. Statistical analysis was performed using the SPSS v26 software package (IBM, Armonk, New York).

## Results

The average duration of the presurgical optimisation programme in the intervention group was 20 (SD 5) days.

### General characteristics of the population

Two hundred eleven patients were included; 135 participated in the presurgical optimisation programme (intervention group), and 76 formed the control group.

Among the participating patients, 58.5% were men. The average age was 65.76 years (SD 11.5). Non-smokers constituted 75.2%. The average BMI was 28.32 (SD 5.38), and NRS 3.71 (SD 1.35). Most participants (88.6%) were cancer patients, and 14.4% received neoadjuvant therapy. Colorectal adenocarcinoma (54.9%) was the most prevalent surgical diagnosis, and laparoscopy was the most frequent intervention (55.3%). The most common comorbidity was cardiac disease (24.8%), followed by type 2 diabetes mellitus (DM) (21.2%) (Table 2).

### Clinical characteristics of the compared groups before the surgery

The percentage of cancer patients was similar in the two groups: 88.8% in the intervention and 88.2% in the control group.

The number of patients with DM was higher in the control group than in the intervention group (27.6% and 19.3%, respectively). The percentage of patients with inadequately controlled DM was higher in the control group (16.6% compared to 5%).

The average NRS score was similar in the two groups (intervention group: 3.82 [SD 1.47]; control group: 3.29 [SD 0.52]) (Table 2).

### Post-surgery clinical characteristics of the compared groups

The frequency of postoperative complications was 25% (*n* = 33) in the intervention group and 52.6% (*n* = 40) in the control group (*p* < 0.001) [OR = 3.4; 95% CI (1.8; 6.2)] (Table 2), while 14.5% (*n* = 19) of patients in the intervention group and 34.2% (*n* = 26) in the control group suffered from remote postoperative complications [OR = 3.1; 95% CI (1.6; 6.2)] (Table 3).

**Table 3:** Post-surgery clinical results for the entire population, and the intervention and control group

		Total *N* = 211	Intervention group *N* = 135	Control group *N* = 76	*P*
	Yes	20 (11.2%)	10 (9.6%)	10 (13.5%)	0.475
Conversion *N* (%)	No	158 (88.8%)	94 (90.4%)	66 (86.5%)	
Complications *N* (%)	Yes	73 (35.1%)	33 (25%)	40 (52.6%)	< 0.001
	No	135 (64.9%)	102 (75%)	36 (47.4%)	
Remote complications *N* (%)	Yes	45 (21.7%)	19 (14.5%)	26 (34.2%)	0.001
	No	166 (78.3%)	116 (85.5%)	50 (65.8%)	
Infectious complications	Yes	46 (21.8%)	19 (14.5%)	27 (35.5%)	< 0.001
	No	165 (78.2%)	116 (85.5%)	49 (64.5%)	
Type of infectious complications *N* (%)	Surgical wound	10 (4.8%)	7 (5.3%)	3 (3.9%)	
	Surgical site	11 (5.3%)	7 (5.3%)	4 (5.3%)	
	Abdominal collections	9 (4.3%)	2 (1.5%)	7 (9.2%)	
	Abdominal abscesses	2 (1.0%)	1 (0.8%)	1 (1.3%)	
	Pulmonary	2 (1.0%)	1 (0.8%)	1 (1.3%)	
	Urological	6 (2,9%)	5 (3.8%)	1 (1.3%)	
	Gynaecological	0	0	0	
	Sepsis	4 (1.9%)	1 (0.8%)	3 (3.9%)	
	Central line	2 (1.0%)	0	2 (2.6%)	
	Bacteraemia	1 (0.5%)	1 (0.8%)	0	
	Phlebitis	0	0	0	
	No	164 (77.3%)	110 (81.7%)	54 (71.1%)	
Pulmonary complications *N* (%)	Infection	3 (1.4%)	1 (0.8%)	2 (2.6%)	0.118
	Atelectasis	7 (3.4%)	6 (4.5%)	1 (1.3%)	
	Effusion	3 (1.4%)	1 (0.8%)	2 (2.6%)	
	Pneumonia	2 (92.8%)	0	2 (2.6%)	
	No	196 (92.8%)	127 (93.9%)	69 (90.9%)	
Abdominal complications *N* (%)	Surgical site	10 (4.8%)	3 (2.3%)	7 (9.2%)	0.125
	Abdominal collections	16 (7.7%)	10 (7.6%)	6 (7.9%)	
	Abdominal abscesses	7 (3.4%)	4 (3%)	3 (3.9%)	
	No	178 (84.1%)	118 (87.1%)	60 (79%)	
Suture dehiscence *N* (%)	Yes	26 (12.4%)	10 (7.5%)	16 (21.1%)	0.005
	No	185 (87.6%)	125 (92.5%)	60 (78.9%)	
Haemorrhage due to surgical stress *N* (%)	Yes	10 (4.8%)	0	10 (13.2%)	< 0.001
	No	201 (95.2%)	135 (100%)	66 (86.8%)	
Wound complications *N* (%)	Seroma	8 (3.8%)	6 (4.5%)	2 (2.6%)	0.411
	Infection	35 (16.8%)	25 (18.9%)	10 (13.2%)	
	No	168 (79.4%)	104 (76.6%)	64 (84.2%)	
Sepsis *N* (%)	Yes	10 (4.8%)	3 (2.3%)	7 (9.2%)	0.030
	No	201 (95.2%)	132 (97.7%)	69 (90.8%)	
Multiple organ failure *N* (%)	Yes	7 (3.4%)	1 (0.8%)	6 (7.9%)	0.006
	No	204 (96.6%)	134 (99.2%)	70 (92.1%)	
Paralytic ileus *N* (%)	Yes	15 (7.2%)	11 (8.3%)	4 (5.3%)	0.410
	No	196 (92.8%)	124 (91.7%)	72 (94.7%)	
Blood transfusion requirements *N* (%)	Yes	32 (15.5%)	13 (9.9%)	19 (25%)	0.014
	No	179 (84.5%)	122 (90.1%)	57 (75%)	
Units of packed red blood cells transfused (SD)		0.42 (1.52)	0.22 (0.80)	0.75 (2.24)	< 0.001
Deaths *N* (%)	Yes	5 (2.4%)	2 (1.5%)	3 (3.9%)	0.360
	No	206 (97.6%)	133 (98.5%)	73 (96.1%)	

*SD* Standard deviation, *N* Number

Suture dehiscence occurred in 7.5% of patients in the intervention group (*n* = 10) and 21.1% (*n* = 16) in the control group; (*p* = 0.005) [OR = 3.3; 95% CI (1.4, 7.7)]. Haemorrhage due to surgical stress happened in 0% in the intervention and 13.2% (*n* = 10) in the control group (*p* < 0.001) [OR = 3.1; 95% CI (2.5; 3.7)] after surgery while sepsis arose in 2.3% (*n* = 3) and 9.2% (*n* = 7); (*p* = 0.03) [OR = 4.5; 95% CI (1.1; 17.8)] of the intervention and control group patients. Multiple organ failure occurred in 0.8% of the subjects (*n* = 1) in the intervention group and 7.9% (*n* = 6) in the control group; (*p* = 0.006) [OR = 11.5; 95% CI (1.3, 97.3)]. The proportion of patients requiring blood transfusion was lower in the intervention group than among the control subjects (90.1% versus 75%, *p* = 0.014) [OR = 3.1 95% CI (1.4, 6.8)]. The programme participants received fewer units of packed red blood cells than the members of the control group (0.22 (0.80) vs 0.75 (2.24), *p* < 0.001) (Table 3).

### Nutritional parameters in the intervention group at the baseline and after 30 days of the presurgical optimisation programme

The levels of albumin, prealbumin, total lymphocyte count, haemoglobin, ferritin, C-reactive protein and total cholesterol improved in the intervention group after 30 days of optimisation (Table 4).

**Table 4:** Laboratory parameters in the intervention group at baseline and after 30 days of the presurgical optimisation programme

Parameter	Start of presurgical optimisation *N* = 135	30 days from the start of presurgical optimisation *N* = 135	*P*
Albumin (g/dl)	2.53 (0.6)	3.51 (0.5)	< 0.001
Prealbumin (mg/dl)	9.1 (6.2)	24.78 (6.5)	< 0.001
Lymphocytes	1.08 (0.6)	1.23 (0.8)	< 0.001
Cholesterol (mg/dl)	144.2 (46.7)	169.76 (45.8)	< 0.001
CRP (mg/l)	29.56 (35.6)	10.46 (18.4)	< 0.001
Haemoglobin (g/dl)	11.82 (1.76)	14.8 (18.5)	< 0.001
Ferritin (mg/dl)	106.75 (113.7)	297.51 (417.8)	< 0.001

The data shown are averages with SD in brackets

*dl* Decilitres, *g* Grams, *l* Litre, *mg* Milligrams, *N* Number, *CRP* C-reactive protein

### Use of healthcare resources after surgery

The intervention group members spent fewer days in the hospital after surgery (8.34 (SD 6.70) vs 11.63 (SD 10.63) days), and there were fewer readmissions within 30 days after the surgery (7.6% vs 19.7%) than in the control group. Five patients in the optimisation programme group (3.8%) had to undergo a reintervention surgery compared to 26 (34.2%) in the control group [OR = 13.5; 95% CI (4.9; 37.1)] (Table 5).

**Table 5:** Use of healthcare resources after surgery by groups compared

		Total *N* = 211	Intervention *N* = 135	Control *N* = 76	*P*
Days of hospital stay^a^, mean (SD)		9.5 (8.4)	8.34 (6.7)	11.63 (10.6)	0.004
Days of hospital stay, median (maximum and minimum)		–	6 (63–2)	8 (69–1)	–
Readmissions *N* (%)	Yes	25 (12.1%)	10 (7.6%)	15 (19.7%)	0.014
	No	186 (87.9%)	125 (92.4%)	61 (80.3%)	
Reintervention *N* (%)	Yes	31 (15%)	5 (3.8%)	26 (34.2%)	< 0.001
	No	180 (85%)	130 (96.2%)	50 (65.8%)	

*SD* Standard deviation, *N* Number

^a^Hospital stay only refers to the primary stay

## Discussion

This study describes the health outcomes for patients who underwent major elective surgery after participating in a presurgical optimisation programme led by a liaison nurse. The results were compared with those obtained for a control group receiving the usual medical care before surgery. Complications, length of hospitalisation and number of hospital readmissions in each group were recorded and compared.

Healthcare professionals working with presurgical optimisation programmes have reported that the implementation is made difficult by organisational complexity (Heil et al. 2022), insufficient time and training (Partridge et al. 2020). Programmes that involve many professionals can be inefficient due to delays in patient treatment resulting from a lack of communication and incorrect referrals (Whiteman et al. 2016). Unlike other national presurgical optimisation programmes developed by the Spanish Multimodal Rehabilitation Group (GERM) (Vía 2021), the model used here requires only two healthcare professionals. Other models employ several specialists (such as haematologists, endocrinologists, cardiologists, pulmonologists, and physiotherapists) and need an average of five visits per patient (Vía 2021). In the protocol carried out at the Infanta Cristina University Hospital of Parla (Madrid) described in this article, the assessment, management, and coordination of patient care is centrally controlled by the liaison nurse in collaboration with the hospital medical internist. Thus, the optimisation process can begin in less than 48 h after adding the patient to the surgery waiting list. In other models, the nurse takes part only in the coordination and follow-up of interventions, and patients’ assessment is carried out by medical specialists (Dana et al. 2022). It has been reported that a healthcare professional acting as a coordinator facilitates implementation (Heil et al. 2022). Empowering a nurse (with adequate training and support) to assume roles historically performed by other medical professionals expedites access to therapies and simplifies the management of complex patients (Carey et al. 2014).

The results obtained here show that presurgical optimisation significantly reduces the frequency of immediate and remote complications after surgery (*p* < 0.001). This is in agreement with another clinical study of patients undergoing major abdominal surgery, whose postoperative complication rate was 31% after preoperative optimisation and 62% in the control group without such a pre-surgery approach (Barberan-Garcia et al. 2018).

It has been shown that surgical patients with anaemia carry an increased risk of postoperative complications and have a higher mortality rate than those without this condition (Bolshinsky et al. 2022). Treating anaemia before surgery reduces the number of postoperative complications and blood transfusions (Blum et al. 2022)(Froessler et al. 2016) and accelerates the recuperation of mobility [s39]. These findings concur with the results of the current study, in which the handling of anaemia as a part of clinical optimisation probably contributed to improved postoperative evolution of the intervention group.

Suture dehiscence and infectious wound complications were less frequent among the preoperative optimisation programme participants than in the control group. This is in agreement with a meta-analysis published in 2021 showing that improving nutritional status and quitting smoking reduce the frequency of wound infection by 29% and 72%, respectively (Perry et al. 2021). Hyperproteic and hypercaloric oral supplementation with 100% lactoprotein serum, leucine, and vitamin D might also be associated with a decrease in the number of postoperative complications (Lawson et al. 2021). This finding concurs with the conclusions of Perry et al. (2021) in a meta-analysis of 10 clinical studies including 643 patients (Perry et al. 2021). The analysis has shown that postoperative complications decreased in the group supplemented with lactoprotein serum (22%) compared to the control group (32%) (Srinivasaraghavan et al. 2022). Leucine is the only branched-chain amino acid that stimulates the mTOR signalling pathway and, thereby, protein synthesis in the muscle (Anthony et al. 2000). Vitamin D is key in lowering the anabolic threshold for postprandial stimulation of muscle protein synthesis by leucine, which contributes to preserving or increasing muscle mass in older patients (Chanet et al. 2017). Thus, it can be argued that the combined administration of these nutritional elements in an oral nutritional supplement helps the recovery of muscle mass and muscular trophism—which is necessary for the early mobilisation of patients after surgery.

The surgical stress response involves a complex interplay of neuroendocrine, metabolic, and inflammatory-immune processes, triggering a catabolic state with the release of growth factors, energy substrates, and inflammatory mediators while suppressing anabolic hormones and causing fluid retention. Enhanced Recovery After Surgery (ERAS) protocols aim to mitigate these effects through multimodal analgesia, early mobilisation, minimally invasive techniques, and early enteral feeding, reducing surgical stress and promoting faster recovery (Choi et al. 2022; Neville et al. 2014). Additional strategies such as reducing preoperative fasting time, carbohydrate loading, and immunonutrition further decrease postoperative complications by addressing metabolic demands and immune function (Chen et al. 2022). Postoperative gastrointestinal (GI) bleeding, an unusual but serious complication of both GI and non-GI surgeries, can arise from surgery-related causes, unrelated causes, or surgical stress exacerbating a pre-existing condition. While minor postoperative bleeding is common and often uncomplicated, significant bleeding, although less common, is associated with high morbidity and mortality (Ghallab 2018). In this study, presurgical optimisation patients showed no haemorrhage compared to the control group, where its frequency was notably high. Presurgical optimisation can significantly reduce surgical stress by addressing various modifiable risk factors before the operation. Key strategies include improving nutritional status, increasing physical fitness, and managing chronic conditions. Adequate nutrition, particularly protein intake, is essential to mitigate muscle loss and support the body's response to surgical stress. Nutritional interventions such as preoperative carbohydrate loading can help reduce postoperative insulin resistance and catabolism (Surgical Optimization Center | Corewell Health n.d.; Hirsch et al. 2021). Physical conditioning through preoperative exercises enhances muscle strength and endurance, which can improve recovery and reduce complications (Sheill et al. 2020). Additionally, comprehensive patient education and mental preparation can alleviate anxiety and ensure patients are better prepared for surgery, contributing to reduced stress and improved outcomes (Surgical Optimization Center | Corewell Health n.d.). These measures collectively help decrease the length of hospital stays, reduce readmissions, and enhance overall postoperative recovery (Hirsch et al. 2021).

Cancer patients who receive oral nutritional supplementation have also shown better clinical evolution, fewer complications, and need fewer health resources than those who have not received such supplementation (Fukuda et al. 2015; Hsu et al. 2021). For example, patients who received a leucine-enriched supplement to accompany a physical exercise programme showed increased grip strength compared to the control group without supplementation (Storck et al. 2020). Another study has compared the recovery of functional capacity of patients in a presurgical optimisation programme with that of a standard care group. The programme consisted of four interventions: high-intensity resistance and strength training, protein-rich nutrition and supplementation, smoking cessation and psychological support. Four weeks after surgery, the average functional capacity of patients in the intervention group (as measured by a 6-min walk test) rose above baseline, while it decreased in the control group (Weimann et al. 2017).

Likewise, the intake of carbohydrate drinks 2 h before surgery stimulates the release of insulin and ghrelin. It reduces the number of catabolic processes, mitigating the transient increase in insulin resistance that elective surgeries usually produce (Noba and Wakefield 2019). High endogenous glucose levels may increase the risk of surgical complications (Jones et al. 2011), prolonging hospitalisation (Kaška et al. 2010; Awad et al. 2013). The European Society for Clinical Nutrition and Metabolism recommends reducing preoperative fasting to 2 h (Weimann et al. 2021); this advice was followed for the intervention group in this study.

Several meta-analyses vary in their conclusions regarding the reduction of hospitalisation time of patients participating in presurgical optimisation programmes. In a meta-analysis of 9 randomised clinical trials, there were no differences between patients who participated in a presurgical optimisation programme and those who did not (Pang et al. 2022). In an umbrella review, all pooled mean differences were consistent with a reduced length of stay in the prehabilitation group, ranging from a reduction of 0.09 to 4.24 days (McIsaac et al. 2022). Lambert et al. (2021) reported a 1.78-day reduction in hospital stays among patients enrolled in presurgical optimisation programmes compared to those receiving standard care (Lambert et al. 2021). These results are similar to those obtained in the current study, where the hospitalisation in the intervention group was reduced by 3.29 days compared to the control group.

Several additional clinical factors can influence the length of hospital stay and post-operative complications. Laparotomy, surgery conversion, poorly controlled diabetes and paralytic ileus are associated with longer length of hospital stay and postoperative complications (Tevis et al. 2015; Tan et al. 2021). These factors could potentially contribute to the differences in hospital stay and complications observed between the intervention and control groups in this study. A higher rate of laparotomy and conversion in the control group may lead to an increase in the length of hospital stay. A meta-analysis showed that hospital length of stay is significantly shorter for patients who undergo laparoscopy compared to those who undergo open surgery (Cirocchi et al. 2017). In a retrospective study of rectal carcinoma patients, conversion was associated with longer postoperative hospital stays (20 days versus 14 days) (Yamamoto et al. 2009). A meta-analysis found a higher incidence of postoperative complications in patients with poorly controlled diabetes (Tan et al. 2021). On the other hand, postoperative ileus is typically associated with a significant increase in the hospital length of stay, yet it occurred less frequently in the control group compared to the intervention group in this study (Iyer and Saunders 2007).

The results of this research must be interpreted in the context of its limitations, common in retrospective-prospective observational studies of usual clinical practice. Thus, a process to randomly assign the medical records to the control group was not followed neither patients in the intervention group were randomly selected. Instead, convenience sampling was used based on the patients on the surgery waiting list, which could affect the probability of being recruited. Moreover, the degree of motivation of a patient voluntarily participating in research can differ significantly from that of other patients. In this study, the patients in the intervention group could have had different motivations to follow the medical recommendations for preoperative optimisation than those in the control group. The size of the intervention group and control group were different. Patients had different diseases requiring surgery with differing burdens. These differences may weaken the comparability of results between groups. A subgroup analysis of individuals sharing, for example, similar nutritional status or comorbidities, both of which are related to subsequent surgical recovery, was not performed. Study variables did not include any risk prediction measurements to allow for risk adjustment of outcomes and a better comparison of balance between groups, thus limiting the reliability of results.

Despite the limitations, the study presents a favourable tendency towards improving clinical outcomes by implementing a presurgical optimisation programme led by a liaison nurse, designed to reduce the number of post-surgery complications and decrease the length of hospitalisation after a major elective surgery. The findings are in accord with data reported by other studies of similar interventions. Although the design of this study has low internal validity, its external validity is relevant because it reflects the usual clinical practice and the value of the interventions that can be carried out in this context. However, the findings from this study need validation through clinical investigations employing a more rigorous design.

## Conclusions

A presurgical optimisation programme described here was led by a liaison nurse. It included hyperproteic and hypercaloric oral nutritional supplementation (100% lactoprotein serum with leucine and vitamin D), a physical exercise program, strengthening of lung capacity and administration of carbohydrate drinks. Psychological and emotional support and health education were provided to reduce presurgical stress. These combined measures decreased the rate of complications and readmissions and reduced hospitalisation times of patients undergoing major elective surgeries. These results need to be corroborated in clinical, comparative studies with a more robust design.

### Supplementary Information

Additional file 1: Supplementary Table S1. Oral nutritional supplementation composition, 100 ml. Supplementary Table S2. Oral nutritional supplementation composition for diabetic patients, 100 ml.

## Data Availability

The datasets of this study are available from the corresponding author upon reasonable request.
