# Supplementary material for: Evaluation of postoperative results after a presurgical optimisation programme
Source: Perioper Med (Lond). 2024 Jul 15;13:73. doi: 10.1186/s13741-024-00430-7 (PMC11247769; doi:10.1186/s13741-024-00430-7)
Supplement: Supplementary file 1 — Additional file 1: Supplementary Table S1. Oral nutritional supplementation composition, 100 ml. Supplementary Table S2. Oral nutritional supplementation composition for diabetic patients, 100 ml. [file 13741_2024_430_MOESM1_ESM.docx]

**Supplementary Tables**

**Supplementary Table 1. Oral nutritional supplementation composition, 100 ml**

**Supplementary Table 2. Oral nutritional supplementation composition for diabetic patients, 100 ml**

**Supplementary Table 1. Oral nutritional supplementation composition, 100 ml**

| Energy | kcal | 200 |
| --- | --- | --- |
| Fats | kcal/g | 8.6 |
| Carbohydrates | g | 20.6 |
| Proteins | g | 10.1 |
| Vitamin D | μg | 5 |
| Moreover, it contains other vitamins, minerals, and trace elements | | |

**Supplementary Table 2. Oral nutritional supplementation composition for diabetic patients, 100 ml**

| Energy | kcal | 104 |
| --- | --- | --- |
| Fats | kcal/g | 3.8 |
| Carbohydrates | g | 11.5 |
| Dietary fibre | g | 2.5 |
| Proteins | g | 4.9 |
| Vitamin D | μg | 1.29 |
| Moreover, it contains other vitamins, minerals, and trace elements | | |
